# Numerical Study on the Static Bending Response of Cracked Wind Turbine Blades Reinforced with Graphene Platelets

**DOI:** 10.3390/nano14242020

**Published:** 2024-12-16

**Authors:** Hyeong Jin Kim, Jin-Rae Cho

**Affiliations:** 1 Department of Mechanical Engineering, University College London, London WC1E7JE, UK; hj.kim.22@ucl.ac.uk; 2 Department of Naval Architecture and Ocean Engineering, Hongik University, Jochiwon, Sejong 30016, Republic of Korea

**Keywords:** cracked wind turbine blade, graphene platelets (GPLs), static bending, deflection, finite element structural analysis, parametric study

## Abstract

With the growing demand for wind energy, the development of advanced materials for wind turbine support structures and blades has garnered significant attention in both industry and academia. In previous research, the authors investigated the incorporation of graphene platelets (GPLs) into wind turbine blades, focusing on the structural performance and cost-effectiveness relative to conventional fiberglass composites. These studies successfully demonstrated the potential advantages of GPL reinforcement in improving blade performance and reducing the blade’s weight and costs. Building upon prior work, the present study conducts a detailed investigation into the static bending behavior of GPL-reinforced wind turbine blades, specifically examining the impact of crack location and length. A finite element model of the SNL 61.5 m wind turbine blade was rigorously developed and validated through comparison with the existing literature to ensure its accuracy. Comprehensive parametric analyses were performed to assess deflection under various crack lengths and positions, considering both flapwise and edgewise bending deformations. The findings indicate that GPL-reinforced blades exhibit reduced sensitivity to crack propagation compared to traditional fiberglass blades. Furthermore, the paper presents a thorough parametric analysis of the effects of crack location and length on blade performance.

## 1. Introduction

The acceleration of global warming and resulting extreme climate changes are widely recognized as some of the most serious challenges humanity faces today. In response, there is growing interest in eco-friendly energy sources like renewable energy, and their share in the global energy mix continues to rise. Simultaneously, various industries are actively developing alternative energy technologies to reduce reliance on conventional fossil fuels. Notably, innovative fuels such as biofuels and electro-fuels (e-fuels) have garnered attention as potential replacements for fossil fuels in the transportation sector, including automobiles, aircraft, and ships [1,2,3]. However, to achieve true zero emissions from production to combustion, these alternative fuels must also utilize renewable energy sources. As a result, demand for renewable energy is expected to increase significantly.

Among the various renewable energy sources, wind energy is projected to account for over 30% of global power generation by 2050 [4]. Consequently, there has been extensive research aimed at maximizing the safety and efficiency of wind turbines. A typical horizontal-axis wind turbine comprises a foundation, tower, nacelle, rotor, and blades. Of these, the blades are critical components that directly influence the turbine’s power generation efficiency and construction costs, making them key elements in terms of both capital expenditure (CAPEX) and operational expenditure (OPEX). As a result, significant research has been devoted to developing new materials to replace the fiberglass composites traditionally used in wind turbine blades. Examples include basalt–carbon hybrid composites [5,6], SiO_2_ and Al_2_O_3_ [7], bamboo [8,9], and carbon fiber [10,11]. Ma and Zhang [12] analyzed the suitabilities of CNT/polymer nanocomposites for wind blade materials in terms of mechanical, fatigue, electrical, thermal, and barrier properties. Dai and Mishnaevsky [13] found that the secondary CNT reinforcements show superior fatigue performances than those without reinforcements through the comparative numerical simulations. Boncel et al. [14] and Buyuknalcaci et al. [15] incorporated nanocomposites, such as carbon nanotubes, into the blades to improve mechanical performance. Pradeep et al. [16] reviewed the research works on advanced materials in fabrication of wind turbine blades and suggested an appropriate material for wind turbine blade applications. Panduranga et al. [17] presented the concept of electrospun polymer nanofibers to enhance mechanical properties of wind turbine blades. Muhammed et al. [7] explored the applicability of nanocomposites for wind turbine materials through material tests and a numerical simulation. Bahaadini and Saidi [18] and Song et al. [19] investigated the aeroelastic behaviors and nonlinear vibration of rotating graphene-reinforced wind turbine blades. In line with these research trends, the authors of this study conducted research to improve the mechanical characteristics, such as bending and vibration, of wind turbine blades by reinforcing them with graphene platelets (GPLs), a cutting-edge nanomaterial. These studies also analyzed the effects of GPL reinforcement in terms of weight reduction and economic feasibility, closely examining the applicability of GPLs in wind turbine blade design [20,21,22].

The above studies on the application of nanocomposites to wind turbines assumed that wind turbines are perfect without any cracks. However, abnormal loading in real operation such as wind-induced dynamic impact may induce micro-cracking [23] within graphene-reinforced wind blades. As is well known, cracking is a critical factor affecting the structural integrity of wind turbine blades. Cracks are initiated by various factors, including cyclic loads and thermal stress, and their propagation can reduce the structure’s stiffness, potentially leading to catastrophic failure. Therefore, thorough crack analysis is essential during both the design and maintenance of all engineered structures. Cracks in wind turbine blades generally fall into two categories: longitudinal and transverse. These cracks most commonly occur at the trailing edge, where the pressure side and suction side shells are bonded together [24]. Wang et al. [25] analyzed the cracking mechanisms in wind turbine blades through finite element analysis and coupon tests, finding that the adhesive bonding the two shells at the trailing edge had lower fatigue strength than fiberglass composites. They identified adhesive failure as the primary cause of transverse cracks. Miao and Chen [26] explored the cracking mechanism and damage characteristics under edgewise bending loads using finite element analysis and observed a rapid increase in transverse cracks once the blade model reached its ultimate strength. Ataya and Ahmed [27] conducted a statistical analysis of crack lengths in approximately 100 wind turbine blades ranging from 100 kW to 300 kW.

Despite these studies, the detailed data on crack behavior in modern wind turbine blades, which are now often manufactured in sizes exceeding 5 MW, remain limited. Even worse, those have been rarely reported for GPL-reinforced wind turbine blades, so this lack of data poses a significant research gap. Although various studies have addressed crack detection [28,29,30,31], natural frequency [32,33], and crack propagation [34] in wind turbine blades, no studies have yet explored the increase in deflection and stress caused by cracks. Therefore, this study, building on previous research conducted by the authors [20,21,22] without considering cracks, aims to quantitatively analyze the structural behavior of cracked wind turbine blades reinforced with GPLs. More precisely, the bending deflection and stress are compared between fiberglass composite-based blades and GPL-reinforced blades when cracks occur to quantitatively evaluate the superior fracture strength of GPLRC wind blades. A fiberglass composite is taken for the comparison because it has been widely adopted for wind turbine blades. The bending characteristics of GPL-reinforced blades are analyzed in greater detail. A finite element model was developed based on the 5 MW SNL 61.5 m wind turbine model to ensure reliability. The study examined flapwise and edgewise deflections—primary deflection directions for blades—under various conditions, including crack location along the blade’s radial direction, crack location relative to the airfoil, and crack length. Based on these analyses, the effects of each parameter on the wind turbine blade were thoroughly evaluated, providing design recommendations for GPL-reinforced wind turbine blades with consideration for potential cracking.

## 2. Finite Element Modeling of Cracked Wind Blades

### 2.1. Blade Modeling

**Figure 1:**
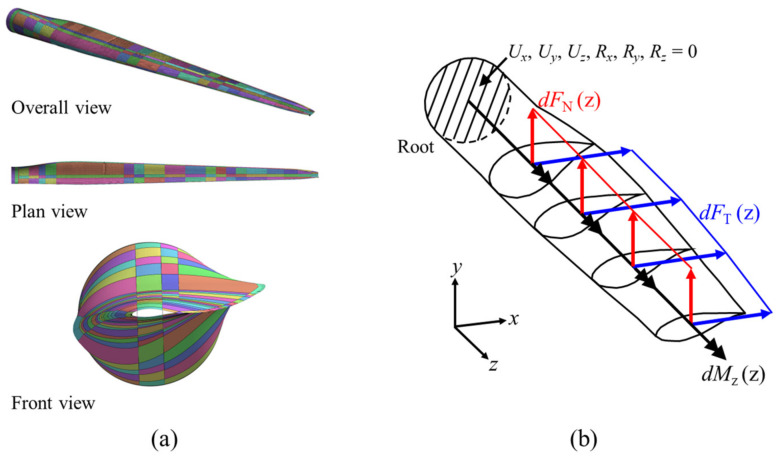
The SNL 61.5 m wind turbine blade model: (**a**) finite element model and (**b**) loading and boundary conditions.

This study developed a finite element model of a fiberglass composite-based blade, using the SNL 61.5 m model—a 5 MW wind turbine blade—as the analysis target [35]. The analysis was performed using midas-NFX 2024R1 (MIDASoft, Inc., New York, NY, USA), a widely used commercial software for finite element analysis [36], with the model created from four-node quad elements. The element size was set to 0.08 m × 0.08 m, consistent with the authors’ previous studies [21,22]. The composite shell element in midas-NFX was used to model the composite laminates within the blade. Detailed information on the model’s geometry and material properties can be found in Resor’s report [35] and the authors’ prior studies [21,22]. Figure 1 illustrates the boundary and loading conditions applied to the finite element model for numerical analysis.

**Figure 2:**
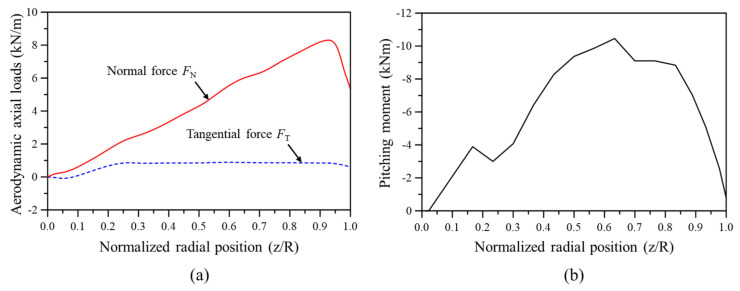
Aerodynamic load distribution calculated using BEMT: (**a**) normal and tangential forces, (**b**) pitching moment.

Wind turbine blades are subjected to a combination of loads, including aerodynamic, inertial, gravitational, and operational forces. Among these, aerodynamic loads significantly influence the blade’s flapwise and edgewise deflections. Therefore, accurately calculating these loads and incorporating them into finite element analysis is crucial for properly evaluating the blade’s bending deformation characteristics. Common methods for calculating aerodynamic loads include blade element momentum theory (BEMT) and computational fluid dynamics (CFD). Due to the complexity and high computational cost of CFD, BEMT is often preferred for large-scale analysis because of its efficiency. In this study, aerodynamic loads acting on the 5 MW wind turbine blade at the rated wind speed (11.4 m/s) were calculated using BEMT, and these results were integrated into the finite element analysis. Figure 2 displays the distribution of normal force, tangential force, and pitching moment as calculated by BEMT. The method for calculating aerodynamic loads via BEMT can be found in the authors’ previous studies [21,22].

### 2.2. Material Modeling

In this study, GPL-reinforced wind turbine blades were implemented by replacing the fiberglass composites in the previously developed blade model with GPL-reinforced composites (GPLRCs). For the GPLRC modeling, the effective material properties were derived from the material properties of GPLs (nanofiller) and epoxy (matrix) based on the linear rule of mixture using Equations (1) and (2).

(1) $$\nu_{eff}=V_{GPL}\;\nu_{GPL}+V_{m}\;\nu_{m}$$

(2) $$\rho_{eff}=V_{GPL}\;\rho_{GPL}+V_{m}\;\rho_{m}$$

where V is the volume fraction of the material, *ν* is Poisson’s ratio, and *ρ* is the density. Subscripts *eff*, GPL, and *m* indicate effective material properties, GPL, and matrix (epoxy), respectively. Note that epoxy was chosen as matrix material because it is most commonly taken as matrix material for both fiberglass composites in wind turbine blades and GPLRCs.

The clustering effect between GPLs is not considered by assuming that GPLs are uniformly dispersed within the matrix. Then, the effective elastic modulus $E_{eff}$ was determined using the Halpin–Tsai micromechanical modeling technique, as shown in Equations (3)–(5). Table 1 provides the material properties of GPLs and epoxy used in the calculation of the effective material properties. Table 2 presents the effective material properties of GPLRCs calculated using the aforementioned modeling technique when $V_{GPL}$ ranges from 1 to 3%. Herein, E represents the elastic modulus, while $V_{GPL}$ denotes the volume fraction of GPLs. The subscript “*eff*” refers to the effective material properties.

(3) $$E_{eff}=\frac{3}{8}\cdot \frac{1+\xi_{L}\eta_{L}V_{GPL}}{1-\eta_{L}V_{GPL}}E_{m}+\frac{5}{8}\cdot \frac{1+\xi_{T}\eta_{T}V_{GPL}}{1-\eta_{T}V_{GPL}}E_{m}$$

(4) $$\eta_{L}=\frac{E_{GPL}-E_{m}}{E_{GPL}+\xi_{L}E_{m}},\;\;\;\eta_{T}=\frac{E_{GPL}-E_{m}}{E_{GPL}+\xi_{T}E_{m}}$$

(5) $$\xi_{L}=\frac{2l_{GPL}}{t_{GPL}},\;\;\xi_{T}=\frac{2w_{GPL}}{t_{GPL}}$$

where L, T, GPL, and m represent the longitudinal direction, transverse direction, graphene platelet, and matrix, respectively. Additionally, $l_{GPL}$, $w_{GPL}$, and $t_{GPL}$ indicate the length, width, and thickness of GPL, respectively. They were set to $l_{GPL}=2.5\;\mathrm{nm}$, $w_{GPL}=1.5\;$μm, and $t_{GPL}=2.5\;\mathrm{nm}$, respectively [19,20].

### 2.3. Crack Modeling

**Figure 3:**
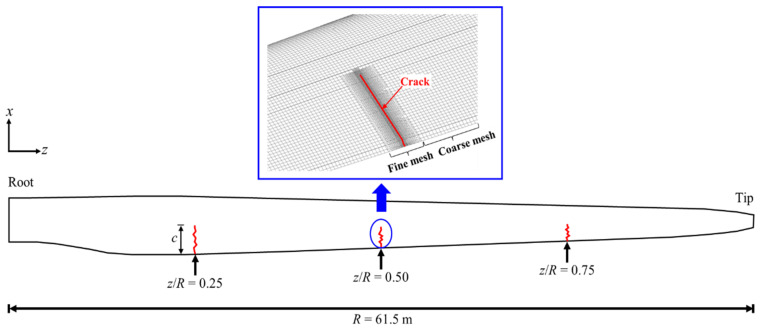
Location of cracks in the wind turbine blade.

In this study, three points with *z*/*R* values of 0.25, 0.50, and 0.75 were selected as crack positions to closely examine the bending characteristics based on crack location, as shown in Figure 3. Here, *R* represents the blade’s radius, and *z* is the coordinate along the radial direction. To ensure accurate analysis results, the elements surrounding the cracks were locally refined according to the gradient h-refinement technique [37], as depicted in the upper part of Figure 3. The smallest size of the elements on the crack was set by 10 mm, which corresponds to 1/16 of the shortest crack length (160 mm) among our analysis cases. And, the size of the element in the transient mesh zone gradually increases with a uniform gradient such that it becomes identical with the element size in the coarse uniform mesh. The cracks were modeled by separating the nodes between elements at the crack locations.

**Figure 4:**
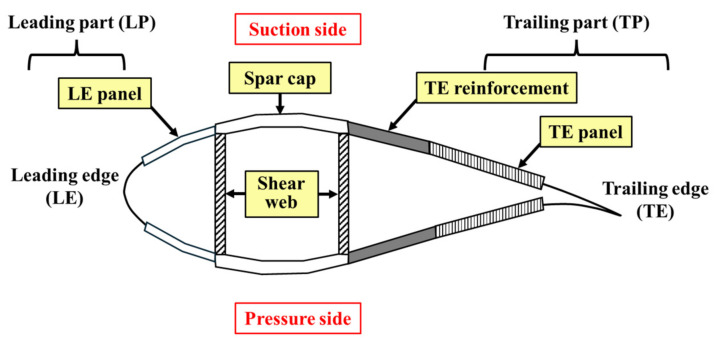
Composing parts of wind turbine blade.

Additionally, the study investigates how bending characteristics change with the occurrence of cracks in various parts of the blade, as illustrated in Figure 4, including the leading part (LP), trailing part (TP), spar cap, and shear web, as well as the crack location along the radial direction of the blade. Cracks in the LP and TP primarily influence edgewise deflection; whereas, cracks in the spar cap and shear web significantly impact flapwise deflection. Consequently, different deflection characteristics were analyzed for each region. 

## 3. Results and Discussion

### 3.1. Validation of the Analysis Model

**Figure 5:**
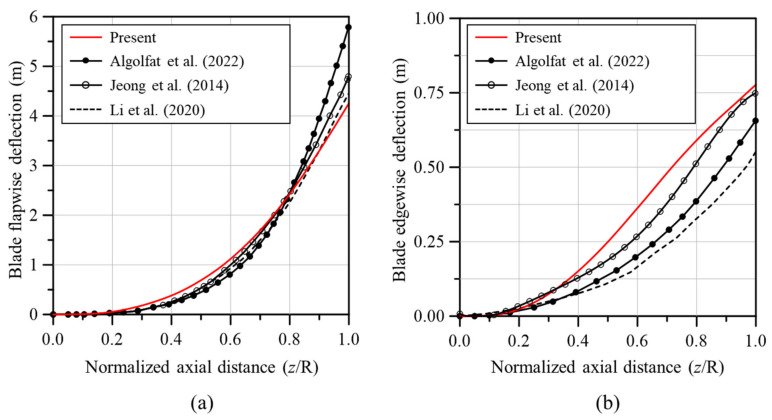
Blade deflection along the blade span [38,39,40]: (**a**) flapwise deflection and (**b**) edgewise deflection.

Before analyzing the effects of cracks on blade bending, the deflection of the intact blade was compared with results from the existing literature to validate the analysis model used in this study. Figure 5 compares the flapwise and edgewise deflection results from this study with those found in the existing literature after applying the aerodynamic loads calculated through BEMT to the analysis model. The flapwise deflection closely mirrored the trends reported in previous studies, affirming the accuracy of the model. Although the edgewise deflection displayed some variation, the results of this study were still deemed reliable, especially when considering the notable discrepancies between other studies in the literature. Moreover, the reliability of the analysis model was further validated by additional factors such as weight and natural frequency, which were thoroughly addressed in previous research conducted by the authors [21,22].

### 3.2. Parametric Investigation

**Figure 6:**
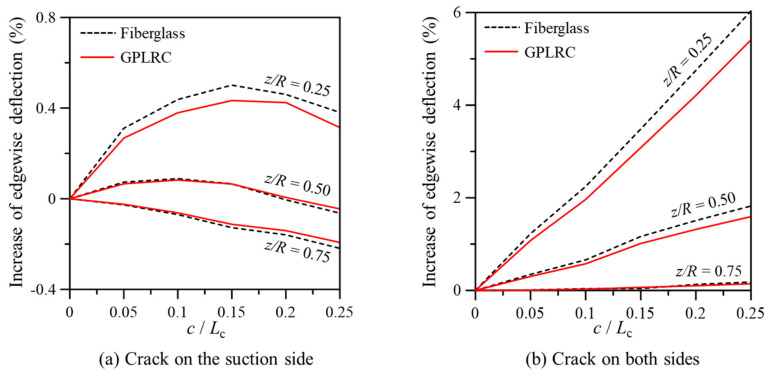
Maximum edgewise deflection in the wind turbine blade with cracks in the LP.

Figure 6 illustrates the maximum edgewise deflection as a function of crack location and length when cracks appear only on the suction side of LP and when cracks occur on both the suction and pressure sides. Here, *c* represents the crack length, and *L_c_* is the chord length of the airfoil cross-section at the crack location. As shown in Figure 6a, the maximum edgewise deflection generally increases as the crack location approaches the fixed end of the blade. However, the deflection characteristics vary with crack length depending on the crack location. At *z*/*R* = 0.25, the maximum deflection increases with crack length but then decreases beyond a certain crack length, likely due to the complex cross-sectional structure of the blade, which differs from simple cantilever beams. At *z*/*R* = 0.75, the maximum edgewise deflection continuously decreases as the crack length increases.

Meanwhile, unlike the case where cracks occurred only on one side (the suction side) of the LP, edgewise deflection exhibited significantly different tendencies when cracks occurred on both the suction and pressure sides, as shown in Figure 6b. Notably, the maximum edgewise deflection was significantly higher compared to the results of Figure 6a. Additionally, the deflection continuously increased at all crack locations as crack length increased. The GPL-reinforced blade exhibited similar deflection characteristics to the conventional fiberglass composite blade, but its stiffness was less affected by the cracks, indicating that GPL reinforcement can help mitigate the reduction in structural integrity caused by cracks.

**Figure 7:**
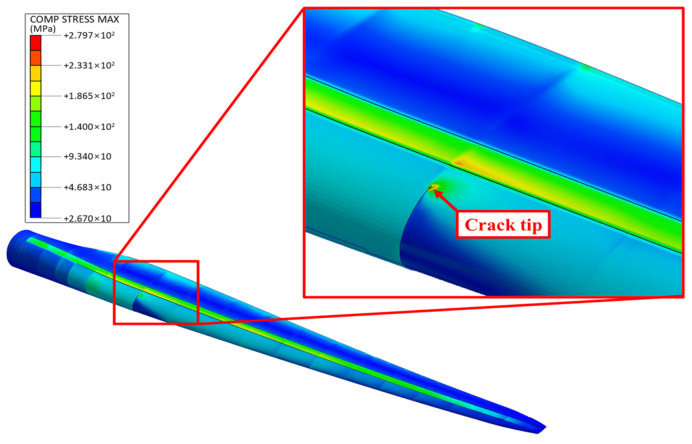
Stress distribution in the GPL-reinforced wind turbine blade with the worst-case crack in the LP.

Figure 7 presents the stress distribution in the GPL-reinforced blade for the scenario with the largest edgewise deflection, which occurred at *z*/*R* = 0.25 and *c*/*L_c_* = 0.25, with cracks on both sides of the LP. The figure shows stress concentration at the crack tip due to geometric discontinuities, with the maximum stress calculated at 279.7 MPa. In addition to the crack region, the spar cap exhibited the highest stress among the remaining parts of the blade.

**Figure 8:**
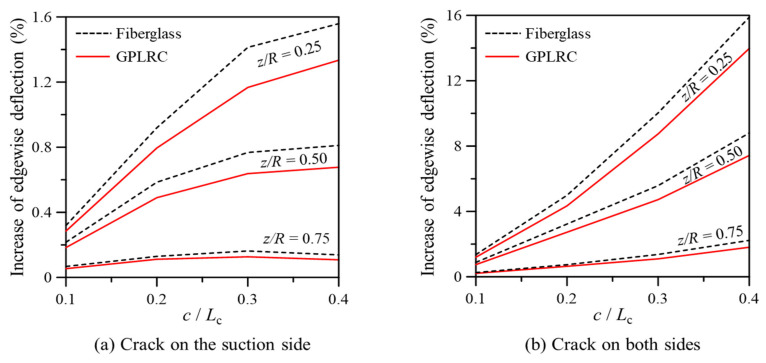
Maximum edgewise deflection in the wind turbine blade with cracks in the TP.

Figure 8 shows the maximum edgewise deflection for cracks in the TP, both when cracks occurred only on the suction side and when they occurred on both the suction and pressure sides. In Figure 8a,b, the maximum edgewise deflection increased as the crack location moved closer to the fixed end, and deflection tended to rise with increasing crack length. When cracks occurred only on the suction side, the increase in edgewise deflection gradually converged as crack length increased. However, when cracks appeared on both sides, the edgewise deflection increased significantly, indicating more severe structural stiffness degradation. This suggests that the uniform changes in crack location on both sides contribute to a more pronounced reduction in stiffness. Additionally, similar deflection trends were observed for both the GPL-reinforced blade and the conventional fiberglass composite blade. However, the GPL-reinforced blade exhibited less stiffness degradation due to the cracks, highlighting its superior resistance to structural damage.

**Figure 9:**
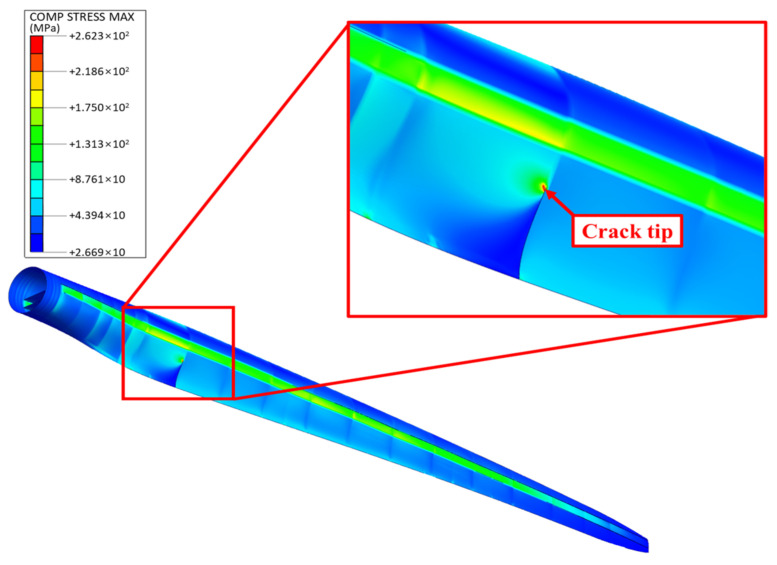
Stress distribution in the GPL-reinforced wind turbine blade with the worst-case crack in the TP.

Figure 9 depicts the stress distribution in the GPL-reinforced blade for the case of the largest edgewise deflection observed in TP cracks, occurring at *z*/*R* = 0.25 and *c*/*L_c_* = 0.40. Similar to Figure 7, stress concentration was seen at the crack tip, with a maximum stress of 262.30 MPa. In the areas other than where the crack occurred, the spar cap exhibited the highest stress, as shown in the results of Figure 9.

**Figure 10:**
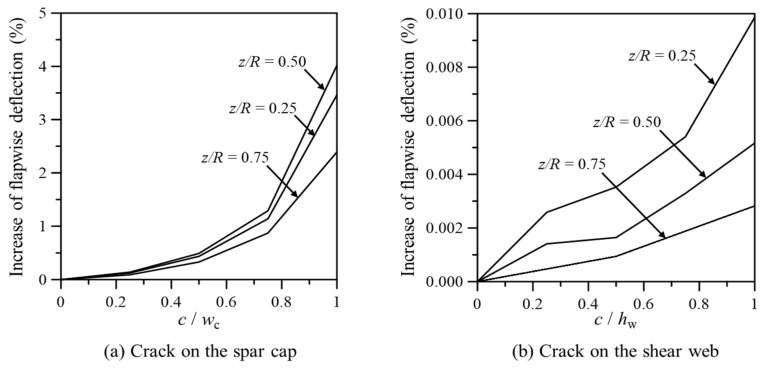
Maximum flapwise deflection in a GPL-reinforced wind turbine blade with cracks on the spar cap and shear web.

Figure 10 illustrates the maximum flapwise deflection according to crack location and length for cracks in the spar cap and shear web of the GPL-reinforced blade. Here, *w_c_* and *h_w_* are the width of the spar cap and height of the shear web, respectively. For cracks in the spar cap, the maximum flapwise deflection occurred when the crack was located at the middle of the blade, rather than near the fixed end, likely due to the complex interplay of blade geometry, material distribution, and load distribution. In the case of shear web cracks, deflection increased as the crack location neared the fixed end, similar to the LP and TP cases, but the increase was minimal (less than 0.01%), indicating that cracks in the shear web have little impact on blade deflection.

**Figure 11:**
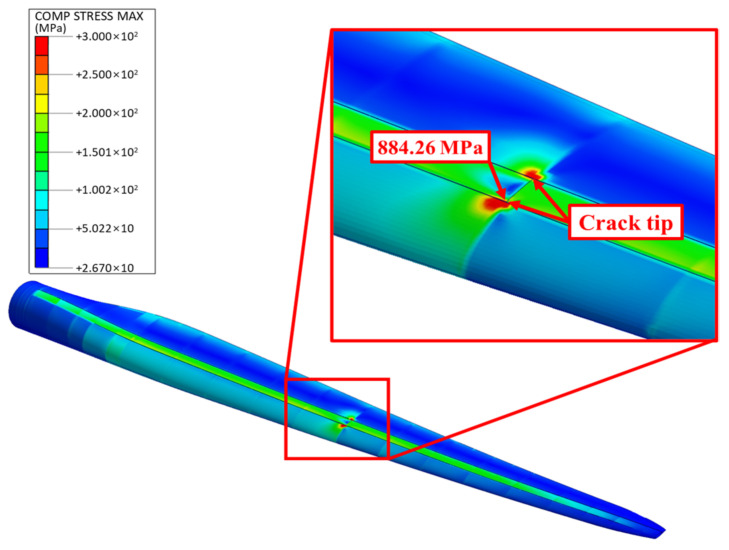
Stress distribution in the GPL-reinforced wind turbine blade with the worst-case crack in the spar cap.

Figure 11 illustrates the stress distribution in the GPL-reinforced blade when the largest flapwise deflection was observed, specifically in the case where cracks occurred in the spar cap (*z*/*R* = 0.50 and *c*/*w_c_* = 1.0). As shown, significant stress concentrations were present at the crack tips on both sides of the spar cap, with the maximum stress reaching 884.26 MPa. This is likely due to the fact that cracks in the spar cap cross-section, which experiences the highest stress (i.e., the greatest resistance to the load), intensify stress concentration in this area. As a result, when cracks occur in the spar cap, they are highly prone to rapid propagation due to the extremely high stress concentration.

**Figure 12:**
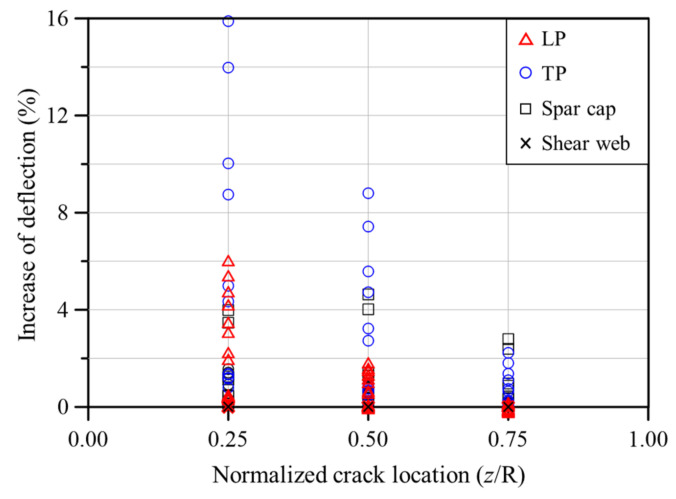
Increase in maximum deflection of wind turbine blades with cracks in different parts.

Figure 12 provides a summary of the deflection analysis. The maximum deflection occurred at the midpoint of the blade in some areas, such as the spar cap, rather than at the fixed end. However, as shown in Figure 12, deflection tends to increase as the crack location moves closer to the fixed end. Notably, the largest deflection was observed when cracks appeared in the TP near the fixed end. Because cracks are most commonly found in the TP, special attention should be given to this area during both the design and maintenance phases.

## 4. Conclusions

In this study, the bending deformation characteristics of cracked wind turbine blades reinforced with GPLs were analyzed in depth. A finite element model was created by referring to the 5 MW SNL 61.5 m blade, and the material modeling of GPLRC was performed using the linear rule of mixtures and the Halpin–Tsai micromechanical technique. Additionally, the aerodynamic loads acting on the wind turbine blade were calculated based on BEMT, and the reliability of the analysis results was verified by comparing the deflection characteristics of the analysis model with results from the existing literature. The analysis model generated the mesh using four-node quad elements and composite shell elements, allowing for an analysis of the bending deformation characteristics of the blade while varying the crack location and length according to the blade radius and cross-section. The main conclusions obtained based on the results of this study are summarized as follows:

- Compared to conventional fiberglass composite-based wind turbine blades, the GPL-reinforced blade exhibited less stiffness degradation due to cracks. Therefore, GPL-reinforced blades are expected to have higher structural safety than conventional blades in the event of cracking.
- Deflection characteristics significantly differed depending on the crack location along the blade radius. In particular, deflection tended to increase as the crack location approached the fixed end of the blade. Under the influence of various complex factors, however, stiffness was reduced most significantly in the case of the spar cap when cracks occurred at the midpoint of the blade rather than at the fixed end.
- While deflection tended to increase with longer crack lengths, the characteristics varied depending on the crack location. When cracks occurred in the TP, deflection continued to increase with crack length; whereas, the rate of deflection increase slowed or decreased beyond a certain crack length in the LP.
- In the case of the shear web, the flapwise deflection increase rate due to cracks was found to be very low (less than 0.01%).
- Stress concentration at the crack tip was observed in all cases. Among the areas not directly affected by the crack, the spar cap exhibited the highest stress levels. In particular, stress concentration was very high when cracks occurred in the spar cap. This situation poses a high risk of rapid crack propagation, highlighting the need for thorough maintenance to prevent cracks in the spar cap.
- All of the deflection analysis results indicated that the stiffness of the blade decreased most significantly when cracks occurred in the TP. This observation aligns with the fact that cracks most frequently occur in the TP, suggesting that more attention needs to be paid to the design and maintenance of TP.

The analysis results confirmed the applicability of GPL-reinforced composites for wind turbine blades and are expected to provide important reference data for developing design optimization strategies that account for crack risks in the future design and maintenance of wind turbine blades.

## Figures and Tables

**Table 1:** Material properties of GPL and epoxy.

Material	E (GPa)	$\nu_{12}$	ρ (kg/m^3^)
GPL	1010.0	0.186	1060
Epoxy	3.0	0.340	1200

**Table 2:** The effective material properties of GPLRCs.

$V_{GPL}$	$E_{eff}$ (GPa)	$\nu_{\mathrm{eff}}$	$\rho_{\mathrm{eff}}$ (kg/m^3^)
0.01 (1%)	11.8	0.338	1199
0.02 (2%)	20.7	0.337	1197
0.03 (3%)	30.0	0.335	1196

## Data Availability

The original contributions presented in the study are included in the article; further inquiries can be directed to the corresponding author.
